# The *Arabidopsis* Pep-PEPR system is induced by herbivore feeding and contributes to JA-mediated plant defence against herbivory

**DOI:** 10.1093/jxb/erv250

**Published:** 2015-06-01

**Authors:** Dominik Klauser, Gaylord A. Desurmont, Gaétan Glauser, Armelle Vallat, Pascale Flury, Thomas Boller, Ted C. J. Turlings, Sebastian Bartels

**Affiliations:** ^1^Zürich-Basel Plant Science Center, University of Basel, Department of Environmental Sciences, Botany, Hebelstrasse 1, CH-4056 Basel, Switzerland; ^2^Université de Neuchâtel, Institute of Biology, Rue Emile-Argand 11, CH-2000 Neuchâtel, Switzerland

**Keywords:** DAMP, herbivory, jasmonic acid, oral secretions, Pep, PEPR, *Spodoptera littoralis*.

## Abstract

A number of plant endogenous elicitors have been identified that induce pattern-triggered immunity upon perception. In *Arabidopsis thaliana* eight small precursor proteins, called PROPEPs, are thought to be cleaved upon danger to release eight peptides known as the plant elicitor peptides Peps. As the expression of some *PROPEPs* is induced upon biotic stress and perception of any of the eight Peps triggers a defence response, they are regarded as amplifiers of immunity. Besides the induction of defences directed against microbial colonization Peps have also been connected with herbivore deterrence as they share certain similarities to systemins, known mediators of defence signalling against herbivores in solanaceous plants, and they positively interact with the phytohormone jasmonic acid. A recent study using maize indicated that the application of ZmPep3, a maize *At*Pep-orthologue, elicits anti-herbivore responses. However, as this study only assessed the responses triggered by the exogenous application of Peps, the biological significance of these findings remained open. By using *Arabidopsis* GUS-reporter lines, it is now shown that the promoters of both Pep-receptors, *PEPR1* and *PEPR2*, as well as *PROPEP3* are strongly activated upon herbivore attack. Moreover, *pepr1 pepr2* double mutant plants, which are insensitive to Peps, display a reduced resistance to feeding *Spodoptera littoralis* larvae and a reduced accumulation of jasmonic acid upon exposure to herbivore oral secretions. Taken together, these lines of evidence extend the role of the *At*Pep-PEPR system as a danger detection mechanism from microbial pathogens to herbivores and further underline its strong interaction with jasmonic acid signalling.

## Highlight

Dysfunction of the Pep-PEPR system and its interplay with JA signalling results in increased plant susceptibility towards herbivore attack indicating that endogenous signalling also contributes to herbivore defence.

## Introduction

Plants use sophisticated perception and signalling systems to detect biotic dangers, such as microbial pathogens or feeding herbivores and, subsequently, to induce an efficient defence response against these threats. In the case of microbial pathogens, several membrane-bound pattern recognition receptors (PRRs) have been characterized that specifically detect conserved microbial structures (referred to as microbe-associated molecular patterns—MAMPs) and eventually trigger a set of defence responses. This mechanism is commonly referred to as pattern-triggered immunity (PTI) (Boller and Felix, 2009; Schwessinger and Ronald, 2012; Macho and Zipfel, 2014).

In the case of herbivorous insects, plants rely on similar detection systems to induce defence signalling and, eventually, herbivore deterrence. The initial recognition of herbivore attack is at least partially achieved by the detection of elicitor compounds in insect oral secretions (Howe and Jander, 2008; Xu *et al.*, 2015) and is potentially mediated by a set of membrane-bound receptors similar to MAMP recognition (Schmelz *et al.*, 2009).

In addition to mechanisms for the detection of exogenous danger, plants also rely on endogenous signalling molecules that are capable of eliciting defence responses (Boller and Felix, 2009; Albert, 2013). Whereas some of these so-called danger-associated molecular patterns (DAMPs), such as cell wall fragments and cutin monomers, are derived from the degradation of the plant cell wall caused by invading pathogens (Sieber *et al.*, 2000; D’Ovidio *et al.*, 2004), others, like the peptides of the systemin family in solanaceous plants, are actively produced by the plant upon the detection of danger (Ryan and Pearce, 2003). Interestingly, systemins have been postulated to be involved in both the deterrence of microbes and herbivores as they have been shown not only to trigger PTI-like responses but also specific defence responses against herbivory. The latter include the biosynthesis of proteinase inhibitors (PI) and the emission of volatile compounds to attract herbivore predators (Ryan and Pearce, 2003; Degenhardt *et al.*, 2010; Sun *et al.*, 2011). However, since the systemin receptor(s) have yet to be fully identified or are under dispute (Holton *et al.*, 2008; Lanfermeijer *et al.*, 2008; Malinowski *et al.*, 2009), the assessment of the biological relevance of systemins to defence signalling has remained difficult.

More recently, a family of endogenous elicitor peptides has been discovered in *Arabidopsis thaliana*, referred to as *At*Peps (Huffaker and Ryan, 2007; Bartels *et al.*, 2013). Like systemins, *At*Peps are small peptides (23–29 amino acids long) derived from the C-terminal ends of larger precursor proteins, the PROPEPs (Yamaguchi and Huffaker, 2011). In contrast to the still contested perception mechanism of systemins, *At*Peps have been shown to be perceived by two membrane-based receptors referred to as PEP-Receptor 1 (PEPR1) and PEPR2 (Yamaguchi *et al.*, 2006, 2010; Krol *et al.*, 2010). Upon *At*Pep perception, both PEPRs trigger PTI-like defence responses reminiscent of the ones elicited by well-known MAMPs, such as flg22 or elf18 (Yamaguchi *et al.*, 2010; Bartels *et al.*, 2013; Flury *et al.*, 2013). Given this similarity between MAMP and *At*Pep-induced responses and the fact that PROPEP/*At*Pep expression is induced upon biotic stress, *At*Peps are believed to function as amplifiers of the initial defence response. In addition they might also be involved in spreading the signal of danger from the damaged or infected area to distal, not yet infected parts of the plant (Boller and Felix, 2009; Yamaguchi and Huffaker, 2011; Ross *et al.*, 2014). A variety modes of amplification of defence signalling by *At*Peps have recently been proposed, either by interacting with defence-related plant hormones (Liu *et al.*, 2013; Tintor *et al.*, 2013; Ross *et al.*, 2014) or by amplifying the production of reactive oxygen species upon previous MAMP detection (Flury *et al.*, 2013; Klauser *et al.*, 2013).

Further support for a role of *At*Peps as amplifiers of defence responses came from the fact that the exogenous application of *At*Peps has been shown to enhance immunity against the hemibiotrophic pathogens *Pseudomonas syringae* (Yamaguchi *et al.*, 2010) and the necrotrophic pathogen *Botrytis cinerea* (Liu *et al.*, 2013). Moreover, the application of *Zm*Pep1, an *At*Pep homologue in *Zea mays* has been shown to induce resistance against *Cochliobolis heterostrophus* and *Colletotrichum graminicola* (Huffaker *et al.*, 2011). However, despite the apparent similarities to systemin, it was only very recently that the exogenous application of *Zm*Pep3 has been shown to induce herbivore defence signalling, including the production of plant volatile emissions, insect deterrent metabolites, and defence-mediating phytohormones, rendering treated maize plants more resistant to the generalist herbivore *Spodoptera exigua* (Huffaker *et al.*, 2013).

However, since only the exogenous application of Peps has so far been shown to induce an increased resistance against herbivore feeding, the contribution of endogenous Pep-signalling to herbivore deterrence has largely remained elusive. Using prom*PROPEP* and prom*PEPR* reporter lines driving a β-glucuronidase (GUS) reporter gene, the expression patterns of both PEPRs, as well as PROPEPs, were investigated here upon feeding by caterpillars of the noctuid moth *Spodoptera littoralis*. Using mutant plants insensitive to *At*Peps, the contribution of endogenous *At*Pep-signalling to herbivore deterrence was also investigated and our observations were linked to specific hormone signalling cascades involved in mediating defence responses against herbivores.

## Materials and methods

### Plant material

*Arabidopsis* plants of the indicated phenotypes were grown individually in small pots at 21 °C with a 10h photoperiod for 4–5 weeks. T-DNA insertion mutants for the *pepr1 pepr2* mutants are in a Col-0 background and were obtained from Birgit Kemmerling (University of Tübingen). The prom*PEPR*::GUS and prom*PROPEP*::GUS reporter lines used are described in Bartels *et al.* (2013).

### Elicitor peptides and insect oral secretions

Peptides of flg22 (QRLSTGSRINSAKDDAAGLQIA) and *At*Pep1 (ATKVKAKQRGKEKVSSGRPGQHN) obtained from EZBiolabs were dissolved in a solution containing 1mg ml^–1^ bovine serum albumin and 0.1M NaCl.

Oral secretions of *Spodoptera littoralis* larvae (obtained from Syngenta Crop Protection, Switzerland) were obtained by gently pushing the forehead region of third and fourth instar larvae as described by Turlings *et al.* (1993). Until use, the oral secretions were stored at –20 °C.

### GUS staining

Plant leaves were either wounded using sterile cork borers, exposed to feeding herbivores as indicated, or treated with 1 μl of *Spodoptera littoralis* oral secretions (by applying two droplets on the upper leaf surface). After 12h, leaves were harvested and the tissue was fixed in ice-cold 90% acetone for 20min, washed with water and then placed in GUS staining buffer (1mM 5-bromo-4-chloro-3-indolyl β-d-glucuronidase (Gold BioTechnology, St Louis, Missouri, USA), 100mM sodium phosphate (pH 7.5), 0.5mM potassium ferricyanide, 0.5mM potassium ferrocyanide, 10mM EDTA, and 0.1% (v/v) Triton X-100) at 37 °C for 12h. Plant tissue was cleared with 70% (v/v) ethanol and photographed using an Olympus SZX12 binocular microscope in combination with an Olympus DP72 camera and the CellSens imaging software (Olympus America, Pennsylvania, USA).

### Gene expression analysis

Total RNA was extracted from *Arabidopsis* leaves using the NucleoSpin RNA plant extraction kit (Macherey-Nagel) and treated with rDNase according to the manufacturer’s specifications. AMV reverse transcriptase together with oligo(dT) primers were used to synthesize cDNA. Quantitative RT-PCR was performed in a 96-well format using a LightCycler® 480 Instrument (Roche). Based on the obtained CT values, normalized expression to the reference gene UBQ10 (AT4G05320) was calculated using the qGene protocol (Muller *et al.*, 2002). The gene-specific primers used were as follows: UBQ10 (AT4G05320) with UBQ_fw (5′-GGCCTTGTATAATCCCTGATGAATAAG) and UBQ_rev (5′-AAAGAGATAACAGGAACGGAAACATAG), PEPR1 (AT1G73080) with PEPR1_qRT-fw (5′-ATTCCTATTGAGATA TGGAAGAG) and PEPR1_qRT_rv (5′-CCTCTTCTAAGCTGC TGTTCAC), PEPR2 (AT1G17750) with PEPR2_qRT_fw (5′-ACCA ATAATTCACCGCGACATC) and PEPR2_qRT_rv (5′-CGCATTT TCTGGTGCAATGTAC), PROPEP1 (AT5G64900) with PP1_qRT_fw (5′-ATCAGATAGACGAAGCGAAG) and PP1_qRT_rv (5′-CTAATTATGTTGGCCAGGAC), and PROPEP3 (AT5G64905) with PP3_qRT_fw (5′-CAACGATGGAGAATCTCAGA) and PP3_qRT_rv (5′-CTAATTGTGTTTGCCTCCTTT).

### Microarray data analysis

Data from two recent microarrays depicting gene expression patterns after either *Spodoptera littoralis* feeding for 8 d (Schweizer *et al.*, 2013) or the exogenous application of *At*Pep2 (Ross *et al.*, 2014) were compared to identify similarly induced genes. This was done by cross-referencing the 50 most strongly up-regulated genes after herbivore feeding to the 1000 most strongly induced genes 2h after the application of 1 μM AtPep2.

### Herbivore feeding assays

Adult plants in the vegetative stage were separately exposed to *Spodoptera littoralis* first instar larvae (10 per plant) for 10 d. Larvae were weighed at the beginning and at the end of the experiment to assess the mass gained, and live larvae were counted to assess survival at the end of the assay. Differences between treatments were then analysed using one-way ANOVA (α=0.05, JMP9). Weight data were square root transformed to meet the assumptions of the model. A total of 15 plants of each of the two *Arabidopsis* lines tested were used.

### Plant hormone analysis

Several leaf discs (90mg fresh weight) were cut from leaves treated by applying 1 μl of insect oral secretions on to the upper leaf surface. Leaf tissue samples were then flash-frozen in liquid nitrogen and stored at –80 °C until hormone level quantification. Hormone extraction and analysis was performed as described by Glauser *et al.* (2013).

### Measurement of ethylene production

For the measurement of ethylene accumulation, three leaf discs of 4–5-week-old plants were harvested using a 5mm cork borer and placed into a 6ml glass vial containing 0.5ml of ddH_2_O, then put back into the growth chamber and left overnight (~16h). Elicitor peptides (1 μM final concentration) and *Spodoptera* oral secretions (0.5% v/v final concentration) were added and vials were closed with air-tight rubber septa. After 4h of incubation at room temperature, ethylene accumulating in the free air space was measured by gas chromatography (GC-14A Shimadzu).

## Results

### The expression of *PROPEP3* as well as both *PEPRs* is induced upon perception of *Spodoptera littoralis* oral secretions as well as herbivore feeding

In maize, individual PROPEPs and Peps were shown to have individual functions. Treatment with ZmPep1 led to an improved resistance against fungal pathogens whereas ZmPep3 application boosted plant defence against herbivores. Similarly, ZmPROPEP1 and ZmPROPEP3 transcription was induced upon treatment with a fungal pathogen or herbivore oral secretions, respectively (Huffaker *et al.*, 2011, 2013).

The involvement of the Pep-PEPR system in fungal resistance has also been shown in *Arabidopsis* and tomato (Huffaker *et al.*, 2006; Liu *et al.*, 2013; Trivilin *et al.*, 2014) but the biological relevance of the observation that a ZmPep3 pretreatment induces anti-herbivore resistance has not been shown due to the lack of PEPR mutants in maize. Thus a switch was made to the model plant *Arabidopsis thaliana* and the generalist herbivore *Spodoptera littoralis* to answer this question.

**Fig. 1.**
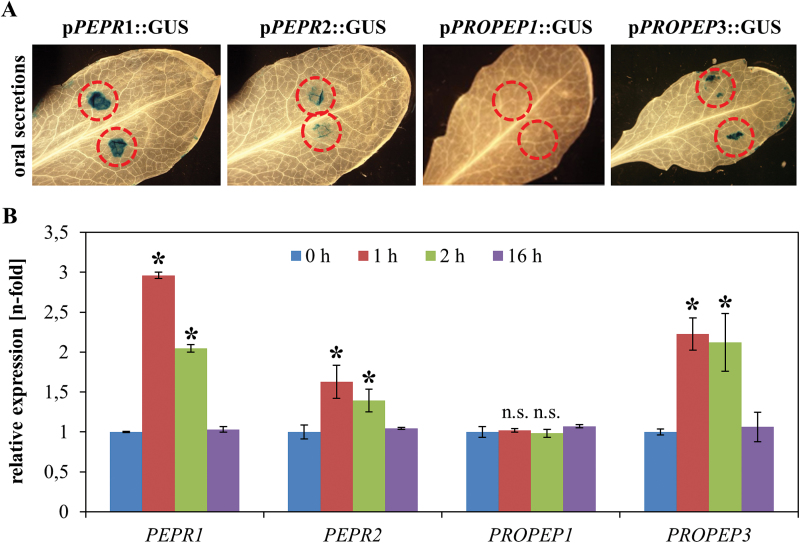
*Spodoptera* oral secretions are sufficient to activate both *PEPR* and *PROPEP3* promoters. (A) 1 μl of *Spodoptera littoralis* oral secretions were pipetted as two small droplets (red circles) onto the leaves of transgenic *Arabidopsis* plants expressing p*PEPR*::GUS, p*PROPEP1*::GUS, and p*PROPEP3*::GUS reporter constructs. After 12h, leaves were detached from the plant, fixed, and stained. For each construct, two independent lines were assessed with similar results. (B) Leaves of *Arabidopsis* Col-0 wild-type plants were treated with 1 μl of *Spodoptera littoralis* oral secretions as described above. After 0, 1, 2, and 16h they were detached from the plant and transcript levels of the respective genes were assessed by qRT PCR. Error bars show ±1 SE of three independent replicates, asterisks indicate significant differences in transcript accumulation compared with untreated plants (*t* test, *P* <0.05).

Transgenic *Arabidopsis* plant lines expressing a β-glucuronidase (GUS) reporter gene under the control of the promoter regions of either *PROPEP1*, *PROPEP3*, *PEPR1*, or *PEPR2* were used as described by Bartels *et al.* (2013) and *Spodoptera littoralis* oral secretions (OS) were applied as two small droplets on to the upper leaf surface of unharmed leaves. In agreement with the up-regulation of *ZmPROPEP3* upon OS detection (Huffaker *et al.*, 2013) *AtPROPEP3* is also induced locally at the site of OS application as detected by GUS staining (Fig. 1A) and via transcript quantification by real-time PCR (Fig. 1B). By contrast, *AtPROPEP1* showed neither a detectable GUS-response (Fig. 1A) nor an increase in transcript abundance after OS application (Fig. 1B). The response of both *PEPR* promoters upon OS perception was also assessed. Similar to *PROPEP3*, both genes are induced upon OS application as shown by local GUS staining (Fig. 1A) as well as by real-time PCR (Fig. 1B). Notably, in contrast to the OS application procedure performed by Huffaker *et al*. which involved scratch-wounding, OS was just pipetted on to the leaf surface and so avoiding wounding and therefore any potential pleiotropic effects of the treatment procedure on our gene expression analysis (Huffaker *et al.*, 2013).

**Fig. 2.**
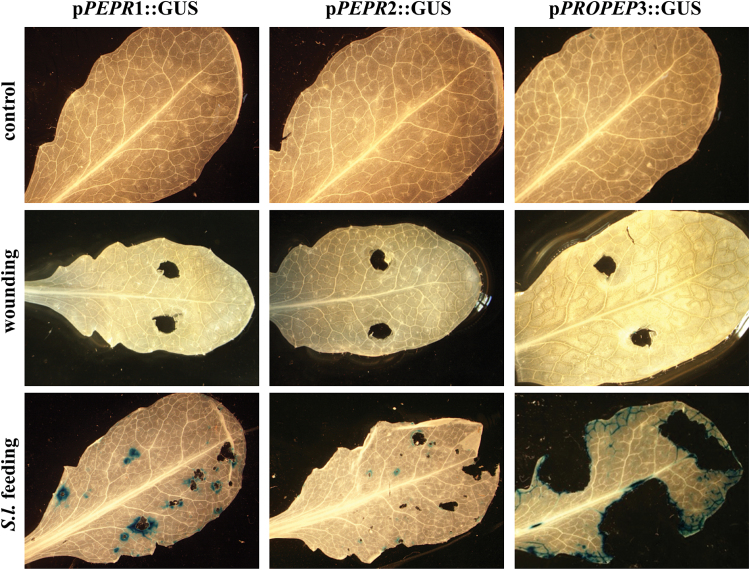
*Spodoptera* feeding strongly induces the promoters of *PEPR1*, *PEPR2*, and *PROPEP3*. Leaves of transgenic *Arabidopsis* plants expressing p*PEPR*::GUS and pPROPEP3::GUS reporter constructs were either wounded using cork borers or exposed to feeding *Spodoptera littoralis* (*S.l*.) larvae. After 12h, the leaves were detached from the plant, fixed, and stained. For each construct, two independent lines were assessed with very similar results.

To assess directly *PROPEP* and *PEPR* gene expression upon herbivore attack, the response of *PROPEP3* and both *PEPRs* to feeding *S. littoralis* larvae were analysed. Similar to the OS application, feeding of *S. littoralis* also strongly activated all three promoters (Fig. 2). The increased activity of the *PEPR* promoters is located directly around areas of herbivore attack and does not extend to unharmed parts of the leaves. In the case of the promoter of *PROPEP3* the detected GUS staining was not limited to the actual feeding sites, but also spread into the leaf veins (Fig. 2). No GUS signal was detected upon wounding the plants by cutting out small leaf pieces using a sterile cork borer (Fig. 2).

**Fig. 3.**
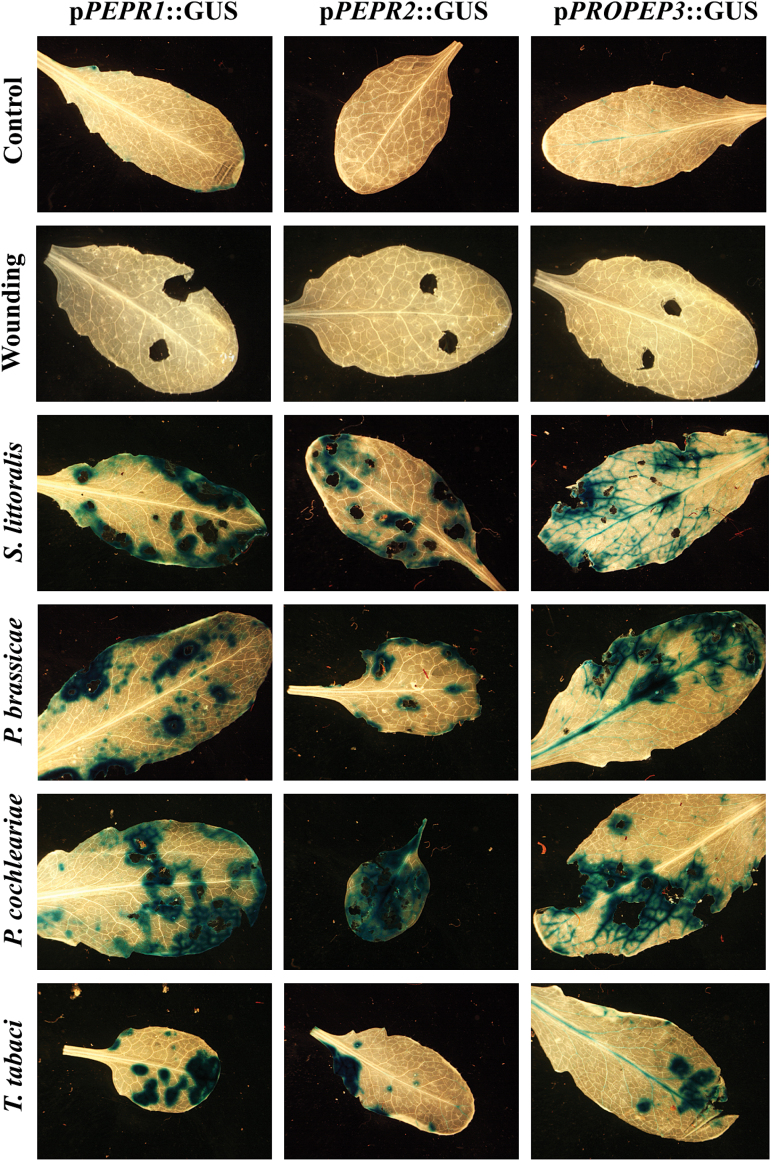
The promoters of *PEPR1*, *PEPR2*, and *PROPEP3* are activated independently of feeding behaviour and specification of the feeding herbivore. Leaves of transgenic *Arabidopsis* plants expressing p*PEPR*::GUS and p*PROPEP3*::GUS reporter constructs were either wounded using cork borers or exposed to feeding insects. After 12h, they were detached from the plant, fixed, and stained. The following insects were assessed (from the top): *Spodoptera littoralis* (generalist, chewing), *Pieris brassicae* (specialist, chewing), *Phaedon cochlearieae* (specialist, chewing), and *Thrips tabaci* (generalist, sucking).

It was notable that the activation of *PEPR* promoters was not limited to feeding of *S. littoralis.* A variety of herbivores were tested on our promPEPR-GUS lines and GUS staining was found in all cases, whereas sterile wounding did not lead to detectable GUS staining (Fig 3). This was independent of the herbivores mode of attack as sucking herbivores such as thrips (*T. tabaci*) were also included, as well as whether the attackers were displaying a generalist (*S. littoralis*) or specialist feeding behaviour (e.g. P. *cochleariae* or *P. brassicae*). Overall, these findings further underline the importance of the Pep-PEPR system for herbivore resistance.

### *Spodoptera littoralis* larvae perform better on *pepr1 pepr2* double mutant plants

**Fig. 4.**
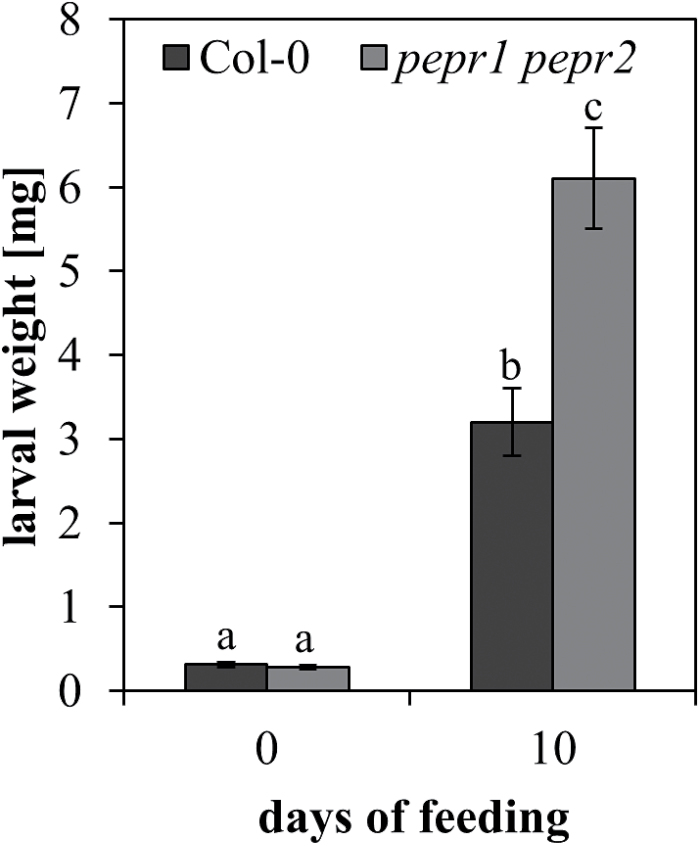
Generalist herbivores perform better on plants impaired in *At*Pep-signalling. Mass of *Spodoptera littoralis* larvae (mean ±1 SE) at the beginning of the experiment (left) and after 10 d of feeding (right) on *Arabidopsis* Col-0 wild-type and *pepr1 pepr2* mutant plants. Letters indicate significant differences between the means (α=0.05, one-way ANOVA, JMP9).

To asses further the indicated importance of the Pep-PEPR system during herbivore challenge, the feeding performance of *Spodoptera littoralis* on *pepr1 pepr2* mutant plants, fully impaired in *At*Pep-signalling (Krol *et al.*, 2010; Yamaguchi *et al.*, 2010), was compared to Col-0 wild-type plants. Ten first instar larvae were placed on each plant for feeding. Ten days later, the larvae were removed and their performance was determined by weight gain. A remarkable difference was found in growth. Larvae feeding on Col-0 wild-type plants reached an average weight of 2.86mg whereas the ones feeding on *pepr1 pepr2* plants showed an average weight of 5.37mg (Fig. 4). Comparing the performance, it was found that *pepr1 pepr2* feeding larvae grew a significant 87% larger than their counterparts on Col-0 wild-type plants (*F*_1,13_=4.82, *P*=0.047). Therefore, the biological relevance of the Pep-PEPR system for herbivore resistance could be proved.

### The response to AtPep perception and *S. littoralis* feeding overlaps in the induction of genes related to jasmonic acid signalling and herbivore resistance

Investigating the mechanism behind the contribution of an activated *At*Pep-signalling system to herbivore recognition and, potentially, deterrence, recently published gene expression data from *Arabidopsis* plants treated either with exogenously applied AtPep2 (Ross *et al.*, 2014) or exposed to feeding *Spodoptera littoralis* larvae (Schweizer *et al.*, 2013) were compared. This analysis revealed several genes which were similarly up-regulated under both circumstances (Table 1). The identified genes encode proteins potentially contributing to direct herbivore deterrence, such as proteinase inhibitors like LTP and TI1 and peroxidases (*PRX52*), transcription factors in defence signalling (FAD-binding proteins) as well as several genes involved in jasmonic acid (JA) biosynthesis and signalling pathways (*JAZ10, LOX3, AOC1*). Intriguingly, similar categories of genes were found to be induced upon the application of *Zm*Pep3 in maize by Huffaker *et al.* (2013), namely proteinase inhibitors (*WIP1, SerPIN*) and genes involved in JA signalling (*AOC, AOS*).

**Table 1.** Genes induced by both the exogenous application of AtPep2 and exposure to feeding Spodoptera littoralis larvae Comparative analysis of data from two recent microarrays depicting gene expression patterns upon either 8 d *Spodoptera littoralis* feeding (Schweizer *et al.*, 2013) or the exogenous application of 1 μM *At*Pep2 (Ross *et al.*, 2014). The genes identified to be similarly induced are listed. Both studies reported *P*-values lower than 0.05 for all genes shown.

Gene annotation	Description	Expression ratio (log_2_) Pep	Expression ratio (log_2_) *Spodoptera*
AT5G05340	PRX52, peroxidase	5.84	3.53
AT5G13220	JAZ 10	4.13	4.83
AT3G44860	FAMT, farnesoic acid methyl transferase	4.11	3.79
AT1G17420	LOX3, lipoxygenase	3.69	3.97
AT5G05600	Oxidereductase, 2OG-Fe(II) oxygenase	2.97	4.57
AT4G20860	FAD-binding berberine family protein	2.88	3.59
AT4G12500	Protease inhibitor (LTP)	2.85	4.27
AT2G38870	Protease inhibitor	2.53	3.55
AT4G37990	CAD8, cinnamyl- alcohol dehydrogenase	2.18	3.86
AT1G74010	Strictosidine synthase	2.09	3.64
AT3G25760	AOC1, allene oxide cyclase	1.97	3.58
AT2G43510	TI1, trypsin inhibitor	1.75	4.79

### PEPR signalling contributes to JA signalling upon herbivore detection

**Fig. 5.**
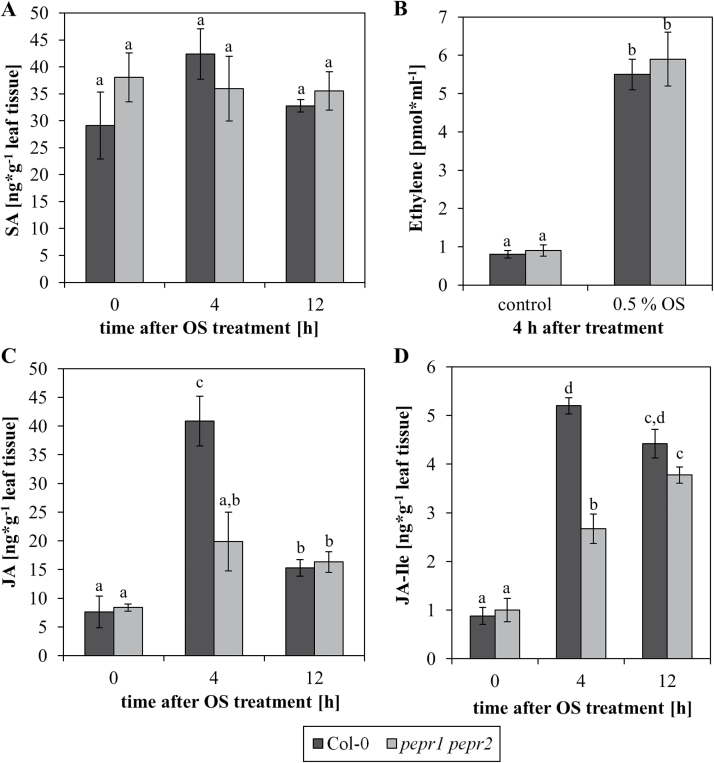
The detection of herbivore oral secretions induces JA biosynthesis in a PEPR-dependent manner. (A, C, D) Leaves of Col-0 and *pepr1 pepr2* double mutant plants were treated by pipetting 1 μl of *Spodoptera littoralis* OS onto the upper leaf surface. After the time indicated, leaves were detached from the plant, flash frozen in liquid nitrogen and the levels of SA (A), JA (C), and JA-Ile (D) were determined by LC-MS. Bars show mean values of eight independent replicates with ±1 SE displayed as error bars. Letters indicate significant differences between the mean values (One-way ANOVA with α=0.05 and *t* test with *P* <0.05). (B) Leaf discs of *Arabidopsis* Col-0 and *pepr1 pepr2* plants were either treated with 0.5% (v/v) OS or without any elicitor (control). Ethylene production was assessed in the headspace 4h after treatment using gas chromatography. Bars show mean values of eight independent replicates with ±1 SE displayed as error bars. Letters indicate significant differences between the mean values (one-way ANOVA with α=0.05 and *t* test with *P* <0.05).

The induction of JA-related genes upon AtPep perception indicates a central role of JA to mediate the induction of herbivore resistance upon PEPR activation. However, the *At*Pep-PEPR system has been shown to interact positively with several hormonal pathways, enhancing defence responses against a variety of pathogens. These include the salicylic acid (SA) (Huffaker *et al.*, 2006; Huffaker and Ryan, 2007; Ross *et al.*, 2014), the ethylene (Huffaker and Ryan, 2007; Liu *et al.*, 2013; Tintor *et al.*, 2013; Ross *et al.*, 2014), and the JA pathways (Huffaker *et al.*, 2006; Huffaker and Ryan, 2007; Ross *et al.*, 2014). To dissect this network further in the specific context of herbivory, the levels of the respective plant hormones were compared between Col-0 wild-type plants and the *pepr1 pepr2* mutant plants before and after the application of herbivore OS (Fig. 5). Upon the perception of OS, the levels of SA did not increase at the time points assessed, with generally no difference being observed between wild-type and mutant plants (Fig. 5A). By contrast, the application of herbivore OS triggered the production of ethylene, with again no detectable difference between Col-0 wild-type and *pepr1 pepr2* mutant plants (Fig. 5B). This, however, was different for the accumulation of JA and its active derivate JA-isoleucine (JA-Ile), where a greater increase of both JA and JA-Ile levels upon OS perception was observed in wild-type Col-0 plants compared with the *pepr1 pepr2* mutant plants (Fig. 5C, D). This difference was most visible 4h after OS application and disappeared when JA levels flattened 12h after treatment, indicating an additional attenuation of the JA response in the mutant. Taken together, the lack of functional PEPR signalling during herbivore perception leads to reduced and/or slower production of JA which probably results in reduced herbivore resistance.

## Discussion

Recently, it was shown that the exogenous application of *Zm*Pep3, an *At*Pep orthologue in maize, induced defence responses against herbivore feeding (Huffaker *et al.*, 2013). Although these findings already suggest a role for Pep-signalling in the plant’s response against herbivores, the biological relevance remained unclear.

With this work, the biological relevance of the Pep-PEPR system in the context of herbivore resistance can now be ascertained by showing that, first, the Pep-PEPR system is induced upon herbivore recognition and, second, that plants lacking a functional Pep-PEPR system are indeed more susceptible to herbivore feeding.

### Herbivore feeding activates the promoters of *Arabidopsis PROPEP3*, but also *PEPR1* and *PEPR2*

In maize, the application of herbivore OS was shown to trigger transcript accumulation of *ZmPROPEP3* (Huffaker *et al.*, 2013). Using quantitative real-time PCR as well as transgenic plants expressing a GUS-reporter gene under the control of the *AtPROPEP3* promoter sequence, these findings could now be confirmed for the model organism *Arabidopsis thaliana.* Intriguingly, a similar expression pattern was also observed for *AtPROPEP1* and *ZmPROPEP1*, both of which are not induced by herbivore oral secretions (Huffaker *et al.* 2013), but respond to the detection of fungal pathogens (Huffaker *et al.*, 2011; Liu *et al.*, 2013). In addition to *AtPROPEP3*, the promoters of *AtPEPR1* and *AtPEPR2*, the two receptors for *At*Peps (Krol *et al.*, 2010; Yamaguchi *et al.*, 2010) also showed rapid activation upon exposure to herbivore OS. This activation was stronger for the promoter of *AtPEPR1* than for *AtPEPR2*, supporting the assumption that *AtPEPR1* is the more important Pep-receptor and reflecting the generally more pronounced expression of *AtPEPR1* as well as the fact that *AtPEPR1* is able to detect all *At*Peps whereas *AtPEPR2* can only detect *At*Pep1 and *At*Pep2 (Krol *et al.*, 2010; Yamaguchi *et al.*, 2010; Bartels *et al.*, 2013). Moreover, based on the report that *At*PEPR2 is important for the repression of *Glutamine Dumper (GUD*) genes and the inhibition of root growth, this receptor might play a more dominant role in the root (Ma *et al.*, 2014). However, in addition to the already mentioned similarities between the regulation of maize *PROPEP*s and *Arabidopsis PROPEP*s, the activation of, specifically, the promoter of *AtPROPEP3* seems to be in line with other recent expression studies, which have shown that, in particular, the expression patterns of *AtPROPEP2* and *AtPROPEP3* are linked to defence signalling (Logemann *et al.*, 2013; Ross *et al.*, 2014). However, despite the apparent central role of PROPEP3 regarding herbivore resistance other PROPEPs and Peps are likely to contribute as well. In *Arabidopsis*, *PROPEP5* is constitutively expressed in leaves and Pep1 has been isolated from unharmed leaves indicating that these PROPEPs and Peps might be released upon damage due to herbivore feeding (Huffaker *et al.*, 2006; Bartels *et al.*, 2013). This would again activate PEPR-triggered defence responses probably contributing to herbivore resistance. Thus, an analysis of PROPEP knock-out mutants could help in understanding the specific contribution of each PROPEP to plant immunity in general and herbivore resistance in particular.

The application of herbivore OS alone, however, constitutes a slightly artificial system as it does not involve the mechanical damage generally occurring upon herbivore feeding (Howe and Jander, 2008). Wounding is known to induce JA accumulation rapidly which would activate anti-herbivore responses and so herbivores make use of elicitors of, for example, microbial origin present in their oral secretions to activate SA signalling and therewith counteract JA signalling (Glauser *et al.*, 2009; Chung *et al.*, 2013). This strategy was shown to be effective in suppressing the induction of wounding-responsive genes upon herbivore feeding using the same model system (*Arabidopsis* and *S. littoralis*) used here (Consales *et al.*, 2012). It was found here that the Pep-PEPR system is induced upon OS perception. This is in line with the robustness of *PROPEP3* induction upon microbial challenges which is not impaired by the dysfunction of either the JA, the ethylene or the SA signalling pathways (Ross *et al.*, 2014). Thus the Pep-PEPR system seems to be immune to a potential perturbation of anti-herbivore signalling by OS elicitors.

Similar to OS application, *Spodoptera* feeding also led to a very strong and local induction of the promoters of both *PEPRs* and *PROPEP3*, whereas sterile wounding alone did not induce the promoters of both *PEPR*s nor *PROPEP3*. It is notable that, previously, activation of the *PROPEP3* promoter was found upon mechanical damage applied with a forceps but this activation was limited to the damaged section of the central vasculature of the leaf and was not detectable in the areas which were treated in this study and where the *Spodoptera* larvae were feeding (Bartels *et al.*, 2013). This indicates a distinct pattern of the Pep-PEPR system activation depending on the danger signal perceived.

Herbivores attack plants with different feeding strategies. Apart from chewing herbivores, such as *S. littoralis*, others, such as aphids and thrips, can nourish themselves from the plant tissue by using stylets either to attack single cells or to suck phloem juice from the plant’s vascular tissue (Howe and Jander, 2008). Most herbivore-derived elicitors have so far been identified in the regurgitant of chewing herbivores (Mithofer and Boland, 2008). However, the activation of the Pep-PEPR system upon feeding of thrips (lacking the production of regurgitant) indicates additional or different modes of herbivore detection, potentially through substances and/or microbes in the attacker’s saliva (Delphia *et al.*, 2007; Chung *et al.*, 2013).

Taken together, the observed local induction of the Pep-PEPR system is not a general response to mechanical damage but a specific and robust response to the perception of herbivore oral secretions and elicitors therein. Unfortunately, given this plethora of potential sources for the HAMP(s) triggering an activation of the Pep-PEPR system, it remains a challenge eventually to identify actual compound(s). Still, the combination of these findings with the fact that the Pep-PEPR signalling system seems to be abundant in all higher plants (Huffaker *et al.*, 2013; Lori *et al.*, 2015) suggests that the Pep-PEPR system is a conserved signalling mechanism for herbivore defence.

### An intact *At*Pep-signalling system is required for full defence responses against herbivores

Several sources have proposed Peps to be considered as endogenous amplifiers of defence responses against a variety of biotic dangers, based on their ability to trigger defence responses and to interact positively with other defence signalling pathways (Boller and Felix, 2009; Yamaguchi and Huffaker, 2011; Macho and Zipfel, 2014). Our comparative analysis of recent transcription profile studies of plants either exposed to exogenously applied *At*Pep2 (Ross *et al.*, 2014) or to feeding herbivores (Schweizer *et al.*, 2013) has led to the identification of a set of similarly induced genes under both conditions, indicating that transcriptional changes upon Pep-signalling include a set of herbivory responsive genes. When combining these findings with the fact that the expression of *PROPEP3* as well as both *PEPR*s is induced by feeding herbivores, it is tempting to expand the aforementioned amplifier theory for *At*Pep-signalling to herbivore deterrence. In agreement with this, feeding *Spodoptera littoralis* larvae perform significantly better on mutant plants lacking a functional *At*Pep-PEPR-signalling system.

### The *At*Pep-system contributes to JA-mediated defence responses

*At*Peps have been suggested to interact with several plant hormone pathways involved in responses to abiotic stress. These pathways include SA (Huffaker *et al.*, 2006), ethylene (Liu *et al.*, 2013; Tintor *et al.*, 2013), and JA (Huffaker and Ryan, 2007; Flury *et al.*, 2013), with ethylene and JA being particularly strongly and positively intertwined with Pep-signalling (Huffaker and Ryan, 2007; Flury *et al.*, 2013; Ross *et al.*, 2014). Furthermore, JA is particularly known to be a major mediator of plant defence responses upon herbivore attack (Howe and Jander, 2008). Aligned with this, our studies revealed that, upon OS detection, both ethylene as well as JA biosynthesis was strongly induced whereas SA levels did not increase.

Moreover, this JA and JA-Ile accumulation was significantly reduced in mutant plants lacking a functional Pep-signalling system. These findings are also aligned with the aforementioned transcriptome analysis, which led to the identification of several genes involved in JA biosynthesis pathways being induced upon both herbivore challenge and treatment with AtPep2 (Schweizer *et al.*, 2013; Ross *et al.*, 2014).

Interestingly, recent studies have shown a positive feedback loop between *At*Pep- and JA-signalling with the application of *At*Peps leading to increased JA accumulation and a functional JA signalling system being required for full-strength Pep-signalling (Flury *et al.*, 2013; Huffaker *et al.*, 2013). In this context, our findings provide additional lines of evidence that support a potential role of the *At*Pep system as an amplifier of JA-mediated defence responses, as shown here in the case of herbivore deterrence.

Both the temporal as well as the spatial resolution of this positive interaction between AtPep- and JA-signalling in the context of herbivore deterrence remain at least partially elusive: First, since PROPEPs are induced by JA and Peps trigger JA accumulation, it needs to be investigated whether the detection of herbivory leads first to an activation of JA signalling, which then induces the Pep-system, or vice-versa. The use of a JA-insensitive mutant might give further insights here but will be complex to analyse due to the positive feedback between both, with not only JA signalling being impaired in JA mutants but also PEPR signalling being reduced which also feeds back on the induction of PROPEP and PEPR expression. Second, as the transcription of both *AtPEPR*s as well as *AtPROPEP3* is induced locally around the site of herbivore detection, it is tempting to speculate that the AtPep-PEPR system is mainly involved in local defence responses. However, Ross *et al.* (2014) showed that, in addition to triggering local defence responses, Pep-signalling is also required for the full activation of systemic defence responses, although as yet only in the context of microbial pathogens. Therefore, apart from the temporal, the spatial resolution of Pep-signalling in the context of herbivore deterrence also requires further investigation and the assays and reporter lines described here could prove helpful tools to investigate these processes further.
